# Loxl2 is dispensable for dermal development, homeostasis and tumour stroma formation

**DOI:** 10.1371/journal.pone.0199679

**Published:** 2018-06-28

**Authors:** Katharina Isabelle Kober, Amparo Cano, Cyrill Géraud, Kalle Sipilä, Seyedeh Atefeh Mobasseri, Christina Philippeos, Angela Oliveira Pisco, Andrew Stannard, Alberto Martin, Fernando Salvador, Vanesa Santos, Michael Boutros, Emanuel Rognoni, Fiona M. Watt

**Affiliations:** 1 Division of Signaling and Functional Genomics, German Cancer Research Center (DKFZ), Heidelberg, Germany; 2 Department of Cell and Molecular Biology, Medical Faculty Mannheim, Heidelberg University, Heidelberg, Germany; 3 Departamento de Bioquímica, UAM, Instituto de Investigaciones Biomédicas "Alberto Sols" CSIC-UAM, IdiPAZ, Madrid, Spain; 4 Centro de Investigación Biomédica en Red, CIBERONC, Madrid, Spain; 5 Section of Clinical and Molecular Dermatology, Department of Dermatology, Venereology and Allergology and European Center for Angioscience, Medical Faculty Mannheim, Heidelberg University, Mannheim, Germany; 6 Centre for Stem Cells and Regenerative Medicine, King's College London, Guy’s Hospital, London, United Kingdom; 7 Department of Physics, King’s College London, Strand, London, United Kingdom; University of Bergen, NORWAY

## Abstract

Lysyl oxidase-like 2 (LOXL2) is a copper-dependent monoamine oxidase that contributes to the remodelling of the extracellular matrix (ECM) by cross linkage of collagen and elastin fibres and has emerged as a potential therapeutic target in cancer and fibrosis. In the skin, LOXL2 is essential for epidermal cell polarity and differentiation. However, its role in the dermis has not been evaluated. We found that *Loxl2* is dispensable for mouse dermal development, maturation and homeostasis, yet affects dermal stiffness. Neither loss of *Loxl2* nor increased *Loxl2* expression affected dermal architecture following treatment with the phorbol ester TPA. Furthermore, *Loxl2* expression did not alter the stroma of DMBA-TPA-induced tumours. We conclude that, although *Loxl2* is expressed in both dermis and epidermis, its function appears largely confined to the epidermis.

## Introduction

Lysyl oxidase‐like 2 (LOXL2) belongs to the lysyl oxidase (LOX) protein family of copper-dependent monoamine oxidases, which has five members, LOX and Lox-like (LOXL) LOXL1, 2, 3 and 4. These proteins are secreted into the extracellular matrix (ECM) and their expression is tightly controlled during development [1]. Deregulated ECM remodelling occurs in pathologic conditions such as cancer and fibrosis [2]. Lysyl oxidases have been associated with multiple extracellular and intracellular functions affecting cell signalling, transcription and translation in tissue homeostasis and disease, and are currently emerging as an attractive therapeutic target in cancer [3]. Lysyl oxidases contribute to remodelling of the ECM by crosslinking collagen and elastin through catalysis of the conversion of ε-amino groups of lysine residues to reactive aldehydes. The latter can react either with other oxidised groups or with lysine residues, leading to a variety of inter- and intra-chain cross-linkages. Accordingly, lysyl oxidases are necessary for the appropriate modification of ECM components and are essential for normal connective tissue development[2–8].

The skin is formed of two layers, the epidermis and dermis, and harbours several appendages, including hair follicles (HF) and sweat glands. The dermis is composed of different fibroblast subpopulations, which give rise to the distinct dermal sublayers: the papillary dermis beneath the basement membrane, the collagen fibre-rich reticular dermis and the dermal white adipose tissue (DWAT), where the pre-adipocytes and adipocytes reside. In addition, subpopulations of fibroblasts envelope HFs (dermal sheath fibroblasts), regulate the HF cycle (dermal papilla fibroblasts) and form the arrector pili muscle (APM) [9].

Aberrant expression of Lox family members has been associated with highly tissue specific developmental defects in mice. Lack of the *Lox* gene has been shown to lead to perinatal death caused by aortic aneurysms, cardiovascular dysfunction, and diaphragmatic rupture [10]. *Loxl1* knockout (KO) mice survive the perinatal period but exhibit disturbed regeneration of elastic fibres in the postpartum intrauterine tract and laxity of the skin, amongst other abnormalities associated with accumulation of the elastin precursor, tropoelastin [11]. In *Loxl2* KO mice, partial perinatal lethality is observed due to heart failure and distended hepatic capillaries. However, in *Loxl2* overexpressing mice only males show a phenotype, which is sterility due to epididymal dysfunction [12]. Loss of *Loxl3* has been shown to cause cleft palate and spinal deformity in mice, coinciding with perinatal lethality [13].

LOXL2 has been shown to participate in ECM remodelling, and also in regulating epithelial-to-mesenchymal transition, epithelial cell polarity and differentiation [14]. Moreover, in a chemical skin carcinogenesis model, *Loxl2* overexpressing mice show an increased tumour burden, whereas *Loxl2* deficient mice exhibit the opposite phenotype. LOXL2 has therefore been identified as an attractive target for cancer treatments [12]. Mechanistically, *Loxl2* negatively modulates epidermal differentiation through repression of *Notch1* promoter activity, leading to a decrease in the steady state activity of the Notch1 signalling pathway. However, the influence of *Loxl2* on the dermis during development and tumour formation is unexplored. Therefore, in this study, we have analysed the dermis of constitutive genetic mouse models, either lacking expression of *Loxl2* or overexpressing *Loxl2*.

## Materials and methods

### Mice

All animal experiments were subject to local ethical approval, have been approved and performed under the terms of a UK Government Home Office license (PPL 70/8474) and Universidad Autónoma de Madrid (UAM) license (Re# CEI‐25‐587). Generation of *Loxl2* knockout (Loxl2-KO) and *Loxl2* knock-in (Loxl2-KI) mice has been described previously [12]. 12-O-Tetradecanoylphorbol-13-acetate (TPA) and 2,4-Dimethoxybenzaldehyde (DMBA)/TPA-protocols were performed as described previously [12]. Briefly, TPA was applied three times at two day intervals during one week at a dose of 12.5 μg. Loss- and gain-of-function mice were bred on different genetic backgrounds [12], and we therefore analysed different controls of the appropriate backgrounds. Since we did not observe any differences between the different controls in the experiments performed, we show only one control in all figures. PDGFRαH2BeGFP [15] mice were bred on a C57BL6/CBA background and male and female mice were used in experiments. Animals were sacrificed by CO_2_ asphyxiation or cervical dislocation. All efforts were made to minimise suffering for mice.

### Human tissue samples

Human foetal tissue was obtained with appropriate ethical approval from the UK Human Developmental Biology Resource. Adult surgical waste skin from written consenting patients under-going plastic surgery was obtained from St George’s University Hospitals NHS Foundation Trust and the study was ethically approved by the National Research Ethics Service Committee UK (HTA Licence No: 12121, REC-No: 14/NS/1073). The age and anatomical location of each tissue sample is listed in (S1 Table).

For total RNA isolation dermal cells were isolated as previously described [16]. Briefly, foetal tissue was minced and digested in trypsin (0.25%) without EDTA (Invitrogen) for 1 hour at 37°C. Human adult skin was cut into 5 mm diameter pieces and incubated with dispase for 1 hour at 37°C; the epidermis was peeled off and discarded and the dermis was digested overnight at 37°C using enzymes from a whole-skin dissociation kit (Miltenyi). The resulting cell suspensions from digested foetal and adult skin were filtered through a 70 μm cell strainer, and centrifuged at 1500 rpm for 10 min at 4°C. The supernatant was removed and the pellet was washed once with PBS at 1500 rpm for 4 min at 4°C. The pellet was resuspended in 350 μl lysis buffer containing 2-mercaptoethanol (Qiagen) for RNA extraction.

### RNA extraction and qPCR

Total RNA was isolated from FACS-sorted GFP^+^ fibroblasts from PDGFRαH2BeGFP mice, isolated fibroblast subpopulations, whole-mounts of OCT-embedded skin from Loxl2-KO, Loxl2-KI and control mice as well as from human skin using the RNeasy kit (Qiagen). Complementary DNA was generated using the QuantiTect Reverse Transcription kit (Qiagen). For the murine samples quantitative PCR (qPCR) analysis of cDNA was performed using qPCR primers (designed with Roche Universal ProbeLibrary Assay Design Center), Roche Universal ProbeLibrary Probes and LightCycler® 480 Probes Master (Roche). qPCR reactions were run on a LightCycler® 480 System (Roche). Actb and 18S rRNA were used as housekeeping genes for normalisation. Please refer to (S2 Table) for primer sequences. Expression levels between different *Lox* family members can be compared, as primer efficiencies were determined previously (S2 Table).

**Fig 1:**
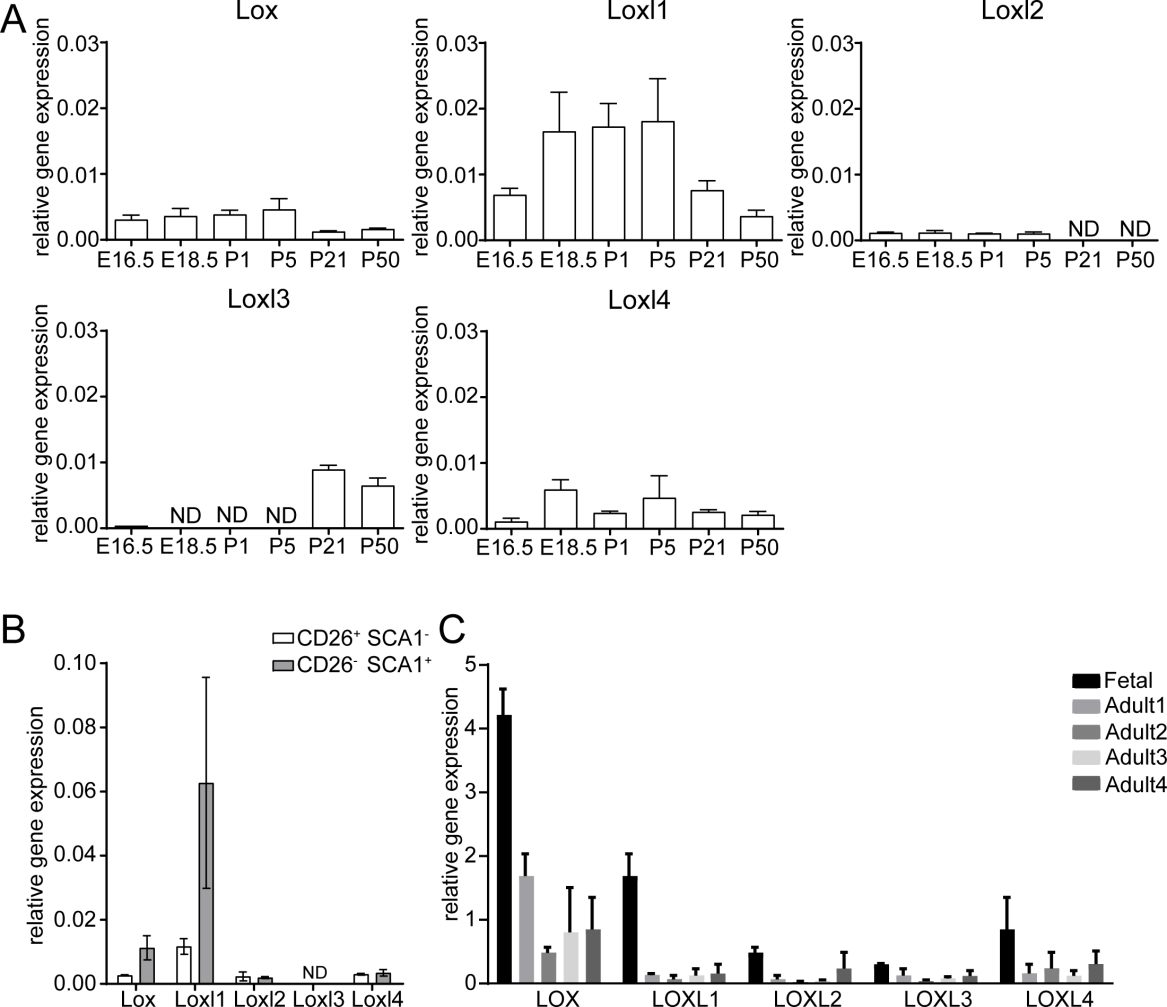
Expression of Lox family members murine and human dermis. A,B: qRT-PCR analysis of Lox, Loxl1, Loxl2, Loxl3 and Loxl4 mRNA expression during mouse development (A) and in FACS-sorted fibroblast subpopulations (papillary fibrbroblasts, CD26^+^ SCA1^-^; reticular fibroblasts, CD26^-^ SCA1^+^) (B) from PDGFRαH2BeGFP mice. C: Expression of LOX family members in human foetal and adult dermis. Details of tissue samples are shown in (S1 Table). ND = no signal detectable.

**Fig 2:**
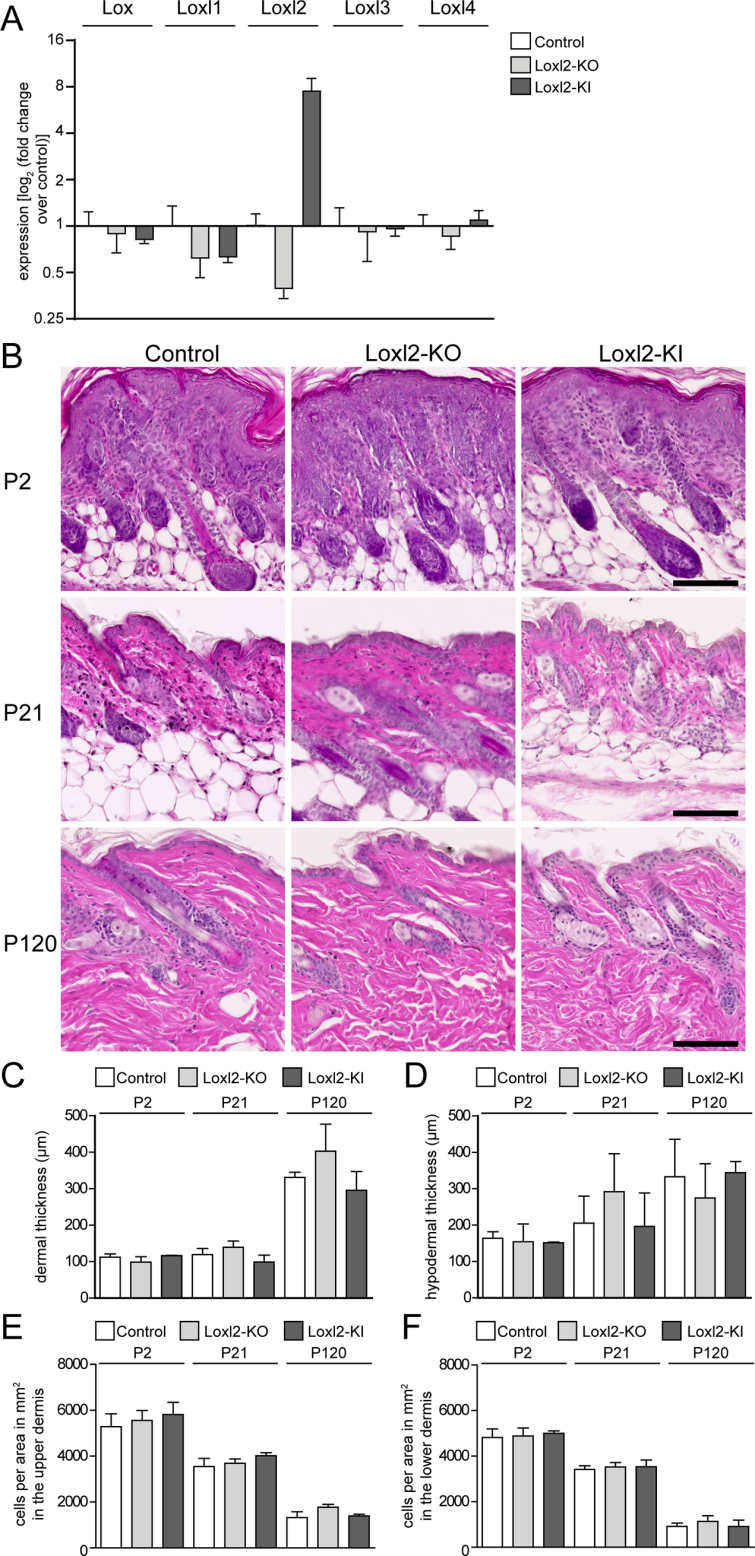
Loxl2 ablation and overexpression are not compensated by Lox family members and do not alter dermal histology, thickness or cell density. A: qPCR analysis of Lox family member mRNA expression in Loxl2-KO and Loxl2-KI mice at P2. (n = 4 for control and Loxl2-KO; n = 2 for Loxl2-KI). B: Hematoxylin and eosin (H&E) staining. Scale bars: 100 **μ**m. C,D: Dermal thickness from basal membrane to hypodermis (C) and of the hypodermis (D). E,F: Total dermal cell number in upper, papillary (E) and lower, reticular dermis (F). (n = 2 for P2 and P120 Loxl2-KI, n = 3 for all others).

For human samples qPCR analysis was performed on the CFX384 Real-Time System (Bio-Rad) using SYBR-Green Master Mix (Life Technologies) and qPCR primers designed with Primer3 (S2 Table). Values were normalised to RPL13A and TBP expression. Analysis of mRNA expression during development (Fig 1) was performed by the delta-Ct method. Target gene expression was normalised to housekeeping genes and the results are the calculated 2^delta-Ct values. For analysis of mRNA expression in Loxl2-KO and Loxl2-KI mice (Fig 2) the calculated 2^delta-delta-Ct values are shown as fold change to control sample of each target gene.

### Genotyping PCR

Genomic DNA from formalin-fixed, paraffin-embedded tissue sections was isolated with the QIAamp DNA FFPE Tissue Kit (QIAGEN) following the manufacturer’s instructions. Primers for PCR genotyping are described in (S3 Table) and have been published previously [12]. L2^+^ is the wild type allele, L2^lox^ the conditional and L2^-^ the knockout allele. R26^+^ is the wild type allele in the ROSA26 locus and R26^L2^ the Loxl2 overexpression allele. PCR reactions were performed using the MyTaq™ HS Red Mix (Bioline) and run on a 2% agarose gel.

### Flow cytometry

Fibroblasts were isolated from PDGFRαH2BeGFP mice as previously described [17–19]. Briefly, back skin of the indicated developmental time points was isolated and incubated in dispase-trypsin solution at 37°C for 1 hour. Neonatal dermis was separated from the epidermis, transferred to 0.25% collagenase in FAD medium, minced and incubated at 37°C for 1 hour. Adult dermis was minced and incubated in FAD medium containing 1.25 mg/ml collagenase type I (Invitrogen), 0.5 mg/ml collagenase type II (Worthington), 0.5 mg/ml collagenase type IV (Sigma), 0.1 mg/ml hyaluronidase IVS (Sigma) and 50 U/ml DNase I (Sigma) at 37°C for 1 hour. Enzyme activity was neutralised by the addition of serum-containing medium. For cell sorting, the dermal cell suspension was passed through a 70 μm cell strainer, washed twice with PBS and labelled with the antibodies (S4 Table) according to standard procedures. 4,6-Diamidino-2-phenylindole (DAPI) was used to exclude dead cells. Labelled cells were sorted on a BD FACSAria Fusion gating for EGFP^+^ and lineage negative (CD45^-^, CD31^-^ and CD324^-^) cells. To obtain fibroblast subpopulations EGFP^+^ and lineage negative (CD45^-^, CD31^-^ and CD324^-^) dermal cells were further sorted into papillary (CD26^+^ SCA1^-^) and reticular (CD26^-^ SCA1^+^) fibroblasts.

### Histochemical stains

Skin samples were fixed in 4% PFA, embedded in paraffin and cut into 8 μm thick sections. Before staining, sections were deparaffinised through a series of xylene (100% Xylene, 100% Xylene; 10 minutes each) and through a series of gradient alcohols to water (100% EtOH, 100% EtOH, 95% EtOH, 95% EtOH, 70% EtOH, 70% EtOH, dH_2_O; 5 minutes each). Rehydrated sections were stained with Mayer's Hematoxylin & Eosin (H&E), Picrosirius Red staining and Herovici staining as described below. Images were obtained with a ZEISS Axio Scan.Z1 slide scanner. Polarised light images of Picrosirius Red stained slides were taken with a ZEISS Axiophot equipped with a polarisation filter.

For H&E staining, slides were stained for 8 minutes in Mayer hematoxylin solution and then washed in running tap water for 10 minutes. Sections were rinsed in distilled water, followed by 10 rinses in 95% alcohol. Sections were counterstained in eosin Y solution for 1 minute, then dehydrated through 95% alcohol and two changes of absolute alcohol (5 minutes each). Following clearing in two changes of xylene (5 minutes each) sections were mounted in DPX mounting medium (Sigma #06522).

Elastica van Gieson staining was performed using a kit from Merck (# 115974) according to the manufacturer's instructions.

Herovici’s staining solution [20] was prepared freshly with 82 ml picric acid (sat. aqu.), 8.2 ml acid fuchsin (1% aqu.), 91 ml methyl blue (0.05% aqu.), 18 ml glycerol and 900 μl Lithium carbonate (sat. aqu.). After deparaffinisation, sections were stained using Weigert’s iron Hematoxylin solution (Sigma) for 4 minutes to label cell nuclei. Slides were then washed for 10 minutes in running tap water and stained in Herovici’s Staining Solution for 2 minutes. Afterwards, slides were rinsed in 1% acetic acid and dehydrated and mounted as described above.

For Picrosirius Red staining, deparaffinised slides were stained with Weigert’s iron Hematoxylin solution as before and then stained in Picrosirius Red staining solution for 1 hour. Afterwards, slides were washed in 0.5% acetic acid (aqu. solution) twice for 2 minutes and dehydrated and mounted as above. When examined by polarised light microscopy, collagen fibres appear red, green or yellow. For quantitation, a colour threshold was set in FIJI and images were converted into a binary image.

### Collagen hybridizing peptide (CHP) labelling

After deparaffinization, sections were blocked with 5% BSA/PBS for 1 hour at room temperature and stained with a biotin-conjugated collagen hybridizing peptide (B-CHP, BIO300, 3Helix) at a concentration of 5 μM overnight at 4°C. According to the manufacturer’s instructions, the B-CHP probe was heated for 5 minutes at 80°C and quenched on ice for 90 seconds, before adding it to the tissue sections. Afterwards, sections were washed three times with PBS and incubated with Streptavidin-AlexaFluor647 (S32357, ThermoFisher Scientific) for 1 hour at room temperature. After washing sections with PBS and incubating them with DAPI (1 mg/ml stock solution diluted 1:10.000 in PBS) for 10 minutes at room temperature, samples were mounted with ProLong® Diamond Antifade Mountant (ThermoFisher Scientific). Confocal microscopy was performed with a Leica TCS SP5 using a 40X objective and the Leica LAS AF software package to stitch multiple 1024×1024 images. For CHP quantification, mean fluorescence signal intensity was measured in the dermis and normalised to the average fluorescence signal of 5 collagen fibres within the selected region of interest. The collagen probe selectively intercalates in the triple helix structure of maturing, remodelling and denatured collagen fibres [21,22].

### Immunostaining of horizontal whole-mounts

Immunostaining was performed on 60 μm cryosections stained as horizontal whole-mounts, as previously described [18,23]. Briefly, skin was fixed in 4% PFA for 10 minutes, washed with PBS and embedded in OCT (Sakura Finetek). Whole-mount sections (60 μm) were cut and immediately placed in PBS to dissolve the OCT. Sections were stained in 300–500μl PB buffer (PBS containing 0.5% skimmed milk, 0.25% cold water fish skin gelatin, 0.5% Triton X-100). Whole-mounts were labelled with primary antibody overnight at 4°C, washed in PBS for 1 hour at room temperature, and incubated with secondary antibodies containing DAPI (1 μg/ml diluted 1:2000) for 2 hours at room temperature. Primary antibodies are listed in (S4 Table). Whole-mounts were mounted on coverslips with glycerol and imaged with a Nikon A1 upright confocal using the NIS-Elements software package to stitch multiple 1024×1024 images.

### Atomic force microscopy (AFM) analysis

AFM (BioScope Resolve™ BioAFM, Bruker) synchronised with optical microscopy (Eclipse Ti-E, Nikon) was used to assess the biomechanical properties of mouse back skin sections. Skin samples were unfixed and cryopreserved in OCT. Prior to AFM measurement, samples were cut to 20 μm thickness and sections were washed with PBS three times and then submerged in 1 ml of fresh PBS at room temperature. For each group, four biological replicates were analysed.

Optical microscopy was used to position the AFM cantilever tip in the dermis at a distance of 50 μm from the epidermal-dermal junction. A silicon nitride PFQNM-LC-A-CAL AFM probe (Bruker) was used, with a spring constant of 0.088 N/m, nominal tip radius of 70 nm, and a semi-included tip angle of 18°. For each replica, three force-volume (FV) measurements were taken, with each FV data set containing 256 (= 16 x 16) measurements over a scan window of 50 μm^2^. Each force-separation measurement was acquired with a ramp size and rate of 6 μm and 0.5 Hz respectively and with a 2 nN trigger force. Analysis of FV data was conducted using the Nanoscope Analysis software package (Bruker). Values of Young’s modulus were extracted from force-separation measurements by fitting to the linearised Sneddon conical model in the force range 0.6 to 1.4 nN with Poisson’s ratio set to 0.5. Statistical significance has been calculated with a Tukey’s multiple comparison.

### Software/ Analysis

All graphs were generated using GraphPad Prism 6 software. Data are presented as means ± standard deviation. Unless stated otherwise, statistical significance was determined by unpaired t-test for biological effects with an assumed normal distribution. ICY software (version Icy 1.9.5.1) was used for image analysis and the spot-detector plugin for identification and quantification of nuclei labelled with DAPI, Ki67 or cCasp3. To quantify DLK1^+^, CD26^+^, LRIG1^+^ and SCA1^+^ cells in the upper and lower dermis, mean fluorescence in the region of interest was determined. Figures were prepared with Adobe Photoshop and Adobe Illustrator (CC2017).

## Results

### Lox family member expression in murine and human dermis

Interrogation of published next-generation RNA sequencing data from mouse skin [24,25] established that, while all Lox family members are predominantly expressed in dermal fibroblasts, dermal papillae cells and melanocytes, *Loxl2* is also expressed by epithelial cells, as previously shown (Part A of S1 Fig) [12]. We confirmed expression of Lox family members by qPCR of sorted GFP+ fibroblasts from PDGFRαH2BeGFP mice. *Loxl2* mRNA was detectable from E16.5 until P5 (Fig 1A), but not in adult fibroblasts at P21 or P50. In contrast, *Lox*, *Loxl1* and *Loxl4* were expressed throughout development from E16.5 until P50, whereas *Loxl3* expression was detected at comparable levels to the other family members only in adult mice (Fig 1A). When Lox family member expression is compared in papillary (CD26^+^ SCA1^-^) and reticular (CD26^-^ SCA1^+^) fibroblast subpopulations at P1, there was little difference in *Loxl2*, *Loxl3* and *Loxl4* expression (Fig 1B). However, *Lox* and *Loxl1* are more strongly expressed in reticular fibroblasts which are associated with high ECM deposition and remodelling activity [16,23].

In human skin LOX family members were more strongly expressed in foetal dermis than adult dermis, consistent with the observations in mouse dermis (Fig 1C). *LOX* showed the highest expression in all samples examined.

### Loxl2 loss- or gain-of-function does not affect dermal development and homeostasis

To evaluate the impact of *Loxl2* expression in dermal development and homeostasis, we analysed loss- and gain‐of‐function transgenic mice that have been previously generated [12]. By qPCR and genotyping PCR, we confirmed *Loxl2* loss- (Loxl2-KO) and gain-of-function (Loxl2-KI) in the skin (Fig 2A and Part B and C of S1 Fig). We detected a pronounced increase in *Loxl2* expression in Loxl2-KI mice and down regulation in Loxl2-KO mice (Fig 2A). We did not observe any significant change in expression of other LOX family members (*Lox*, *Loxl1*, *Loxl3* and *Loxl4*) in the skin of Loxl2-KO and Loxl2-KI mice (Fig 2A). When we analysed skin from neonatal (P2) and adult mice (P21 and P120) by H&E staining, we did not observe any differences in dermal thickness and cellularity between control, Loxl2-KO and Loxl2-KI mice (Fig 2B–2F). In addition, macroscopic observation did not reveal any differences in HF development, growth and cycling. When we scored cell density separately in the upper (Fig 2E) and the lower (Fig 2F) dermis, we found that in all three genotypes the upper dermis had the highest cell density at P2 and that both upper and lower dermal cell density decreased with age, as previously reported [26]. Moreover, *Loxl2* gain- or loss-of-function did not affect the percentage of apoptotic and proliferative cells in the dermis, as measured by cleaved Caspase 3 (cCasp3) and Ki67 immunostaining, respectively, of back skin whole-mount sections (S2 Fig).

**Fig 3:**
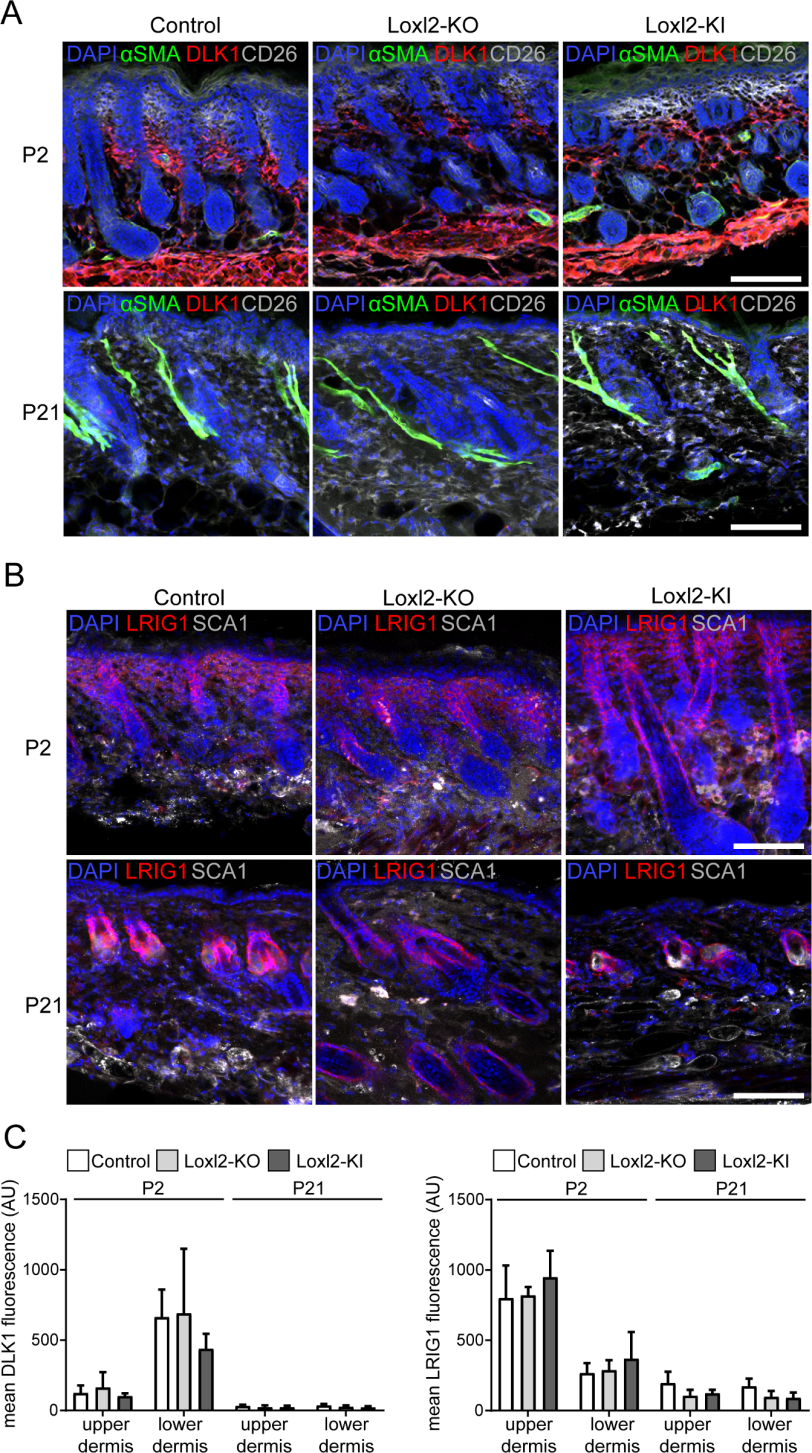
Fibroblast subpopulations are not affected by Loxl2 deletion or overexpression. A: Immunostaining for α-SMA (green), DLK1 (red) and CD26 (white). B: Immunostaining for LRIG1 (red) and SCA1 (white). Nuclei are labelled with DAPI (blue). Scale bars: 100 **μ**m. C: Mean immunofluorescence quantification of DLK1 (left panel) and LRIG1 (right panel) in the upper and lower dermis (n = 2 for P2 Loxl2-KI, n = 3 for all others). Data are means ±SD. Note the high expression of DLK1 in the lower, reticular dermis and LRIG1 in the upper, papillary dermis at P2, which are down regulated with age.

We have previously shown that at E16.5 multipotent dermal fibroblasts differentiate into papillary, upper dermis (CD26^+^; LRIG1^+^) and reticular, lower dermis (SCA1^+^; DLK1^+/-^) fibroblasts [23,26]. The former give rise to cells of the papillary layer, dermal sheath, dermal papilla and APM, the latter form the reticular dermis and further differentiate into pre- and mature adipocytes. With age, DLK1 and LRIG1 expression are highly down regulated, whereas CD26 and SCA1 expression expand throughout the dermis. When we compared neonatal (P2) and adult (P21) skin sections of control and mutant mice, there was no obvious difference in fibroblast subpopulation marker expression and distribution (Fig 3A–3C and Part A and B S3 Fig). Furthermore, the APM (highly positive for α-SMA) started to form at P2 and was fully developed at P21 in control and mutant mice (Fig 3A). Therefore, *Loxl2* loss- or gain-of-function did not influence dermal development, architecture and fibroblast subpopulation distribution.

### Loxl2 overexpression affects collagen remodelling and dermal stiffness

**Fig 4:**
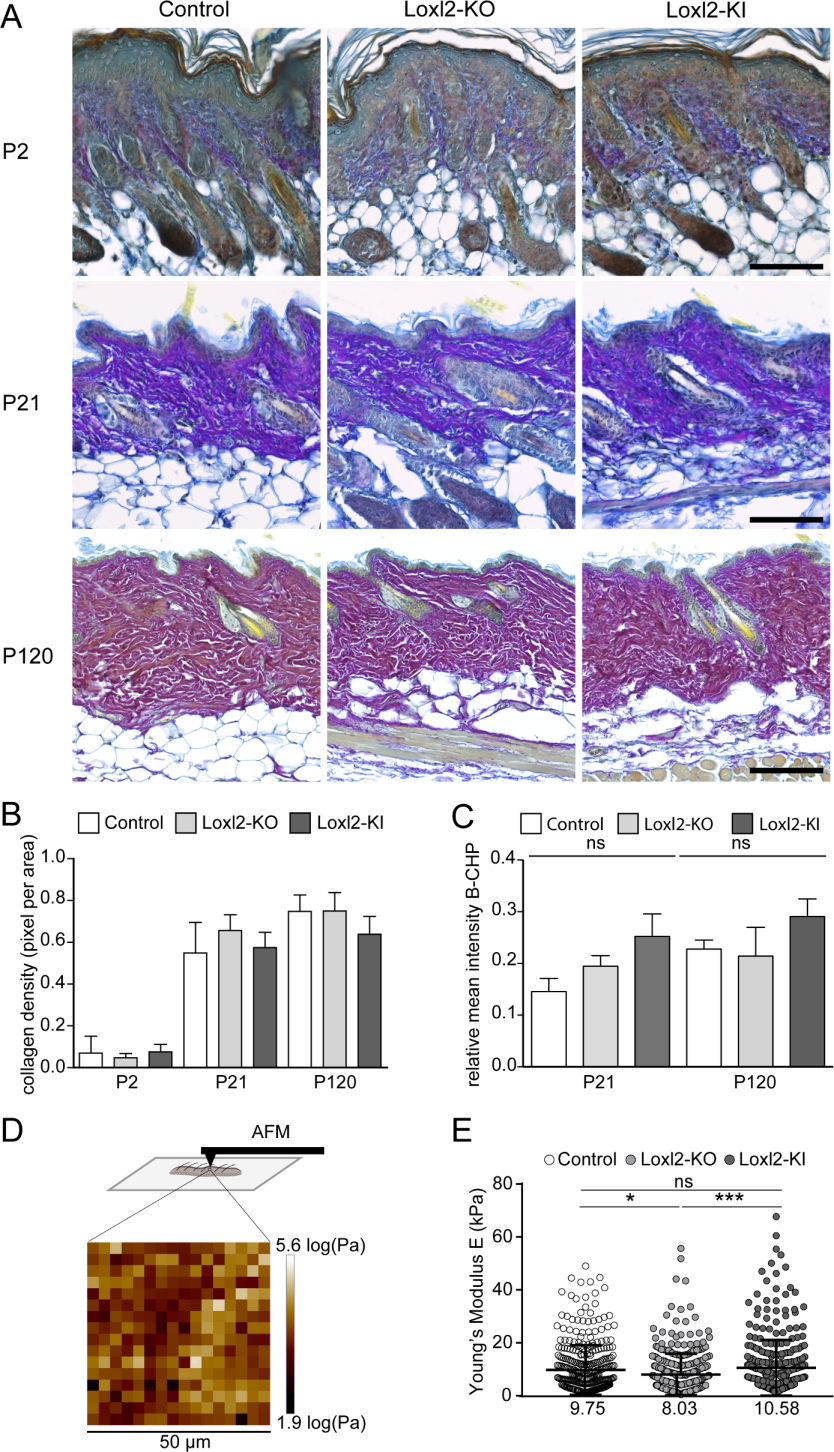
Dermal stiffness but not collagen maturation is altered in the dermis of Loxl2-depleted and overexpressing transgenics. A: Herovici staining, enabling visualisation of immature collagen fibres (blue) and mature collagen (pink/purple). B: Collagen density quantification of Loxl2-KO, Loxl2-KI and control skin samples at the indicated postnatal ages. Collagen density values were calculated as collagen pixel per total tissue. C: Quantification of B-CHP signal. The data shown are means ± SD; n = 7 (Control), n = 5 (Loxl2-KO), n = 2 (Loxl2-KI) at P21 and n = 4 (Control), n = 6 (Loxl2-KO), n = 2 (Loxl2-KI) at P120. ns; not significant. D,E: AFM measurments of P21 old dorsal dermis. Experimental strategy with a representative 50 **μ**m^2^ scan area showing the Young’s Modulus *E* distribution (D). Quantification of Young’s modulus *E* distribution in control, Loxl2-KO and Loxl2-KI dermis at P21. The mean is shown below (n = 4 biological replicates per genotype with 3 scan areas per sample).

To delineate the effect of *Loxl2* loss- or gain-of-function on collagen architecture and deposition, we performed Herovici staining and Picrosirius Red staining. Throughout dermal maturation from P2 to P120, the content of mature, highly-crosslinked collagen accumulated, as shown by increased pink/purple Herovici staining in the P120 dermis (Fig 4A). Immature collagen content, indicated by blue staining, was higher in P2 and P21 samples and decreased over time (Fig 4A). In Picrosirius Red stained sections, collagen bundles appear green, red or yellow under polarised light (Part A of S4 Fig). To quantify total collagen density, we converted the total collagen signal into black pixels in a binary image, revealing how total dermal collagen increased during dermal maturation, coinciding with the increasing thickness of the dermal layer (Fig 4B and Part B of S4 Fig). Neither Herovici nor Picrosirius Red staining revealed any significant differences between control and mutant mice.

To assess differences in collagen fibre structure, we performed staining with B-CHP, which intercalates into accessible collagen triple helixes in maturing, remodelling and denatured collagen fibres [21,22]. With age, B-CHP signal intensity increased, coinciding with increasing thickness of the dermis and collagen maturation (Fig 4C and Part C of S4 Fig). Based on the quantification of B-CHP, we detected a slight increase in Loxl2-KI mice; however, this was not statistically significant (Fig 4C). To investigate whether *Loxl2* gain- or loss- of function affected dermal stiffness, we performed AFM measurements on P21 old dorsal dermis (Fig 4D and 4E). The mean Young’s modulus *E* was significantly increased in Loxl2-KI and decreased in Loxl2-KO back skin, indicating an effect of *Loxl2* on skin stiffness.

In summary, although we did not observe any major differences in collagen architecture, deposition and remodelling between controls and *Loxl2* gain- or loss-of-function animals during dermal maturation, *Loxl2* did affect dermal stiffness.

### Loxl2 loss- and gain-of-function do not affect the dermal TPA response

**Fig 5:**
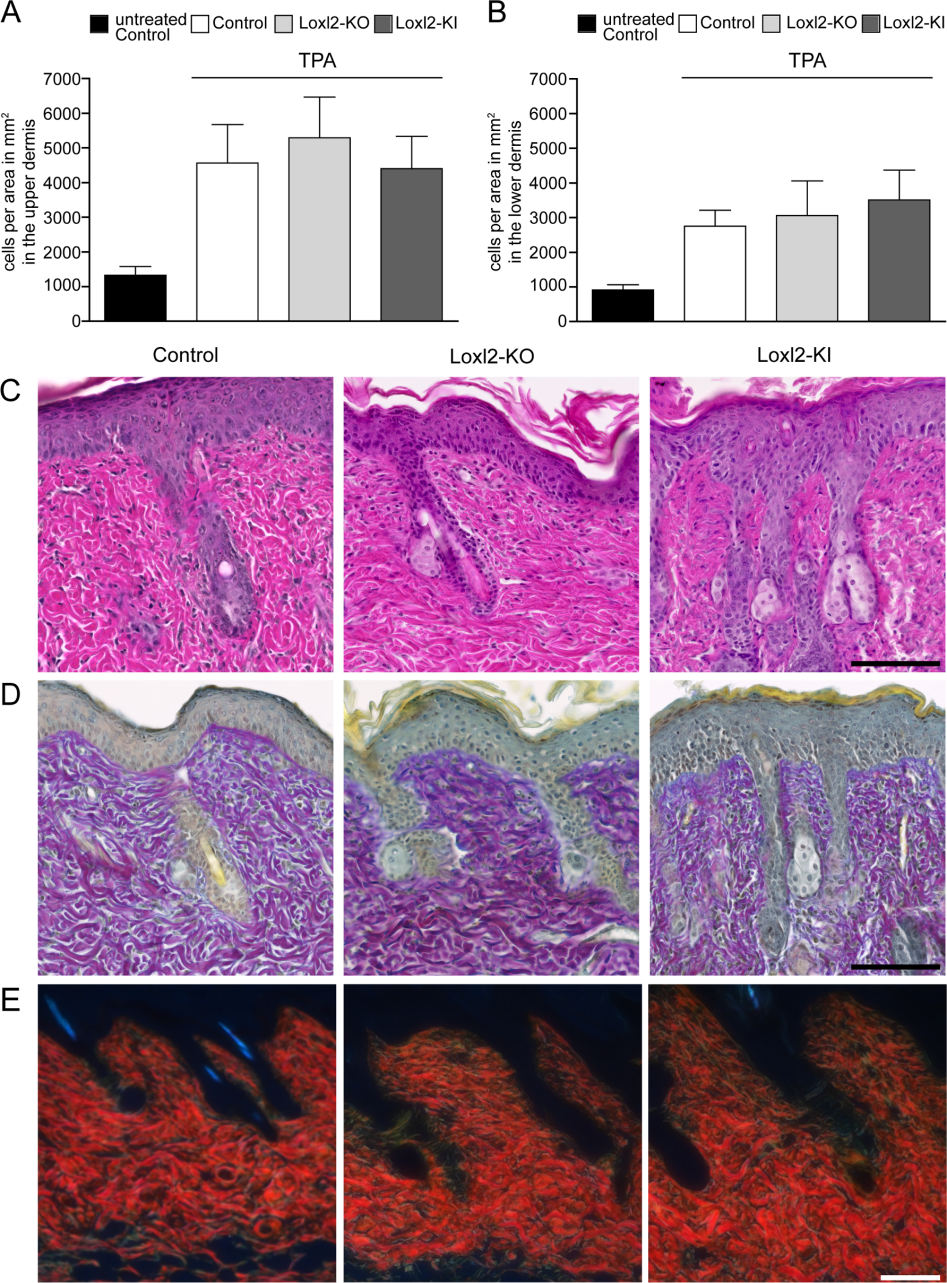
Loxl2 deletion or overexpression do not affect skin response to TPA. A,B: Quantification of dermal cells in the upper, papillary (A) and lower, reticular (B) dermis upon TPA treatment. The data shown are means ± SD; n = 2 (untreated control), n = 6 (Control TPA), n = 2 (Loxl2-KO TPA), n = 3 (Loxl2-KI TPA). C: Hematoxylin and eosin (H&E) staining. D: Herovici staining. E: Picrosirius red staining visualised in polarised light. Scale bars: 100 μm.

To investigate a role of *Loxl2* in pathological skin conditions, we analysed the dermis of control and mutant mice upon treatment with TPA, which is known to stimulate epidermal proliferation and skin inflammation [27,28]. After 7 days of TPA treatment we detected a comparable increase in dermal cell density in the upper and lower dermis (Fig 5A and 5B) and a pronounced epidermal hyperplasia (Fig 5C) in control and mutant mice compared to untreated controls. To delineate the effect of *Loxl2* loss- or gain-of-function on collagen architecture, we performed Herovici and Picrosirius Red staining. In all TPA-treated skin samples, the dermis close to the basement membrane stained slightly blue with Herovici (Fig 5D), indicating the presence of immature and/or remodelling collagen fibres. Picrosirius Red staining revealed high compaction of collagen fibres on TPA treatment (Fig 5E). None of the dermal effects of TPA differed between *Loxl2* mutants and control animals.

### The stromal microenvironment of DMBA/TPA-induced tumours is not altered upon Loxl2 loss- or gain-of-function

**Fig 6:**
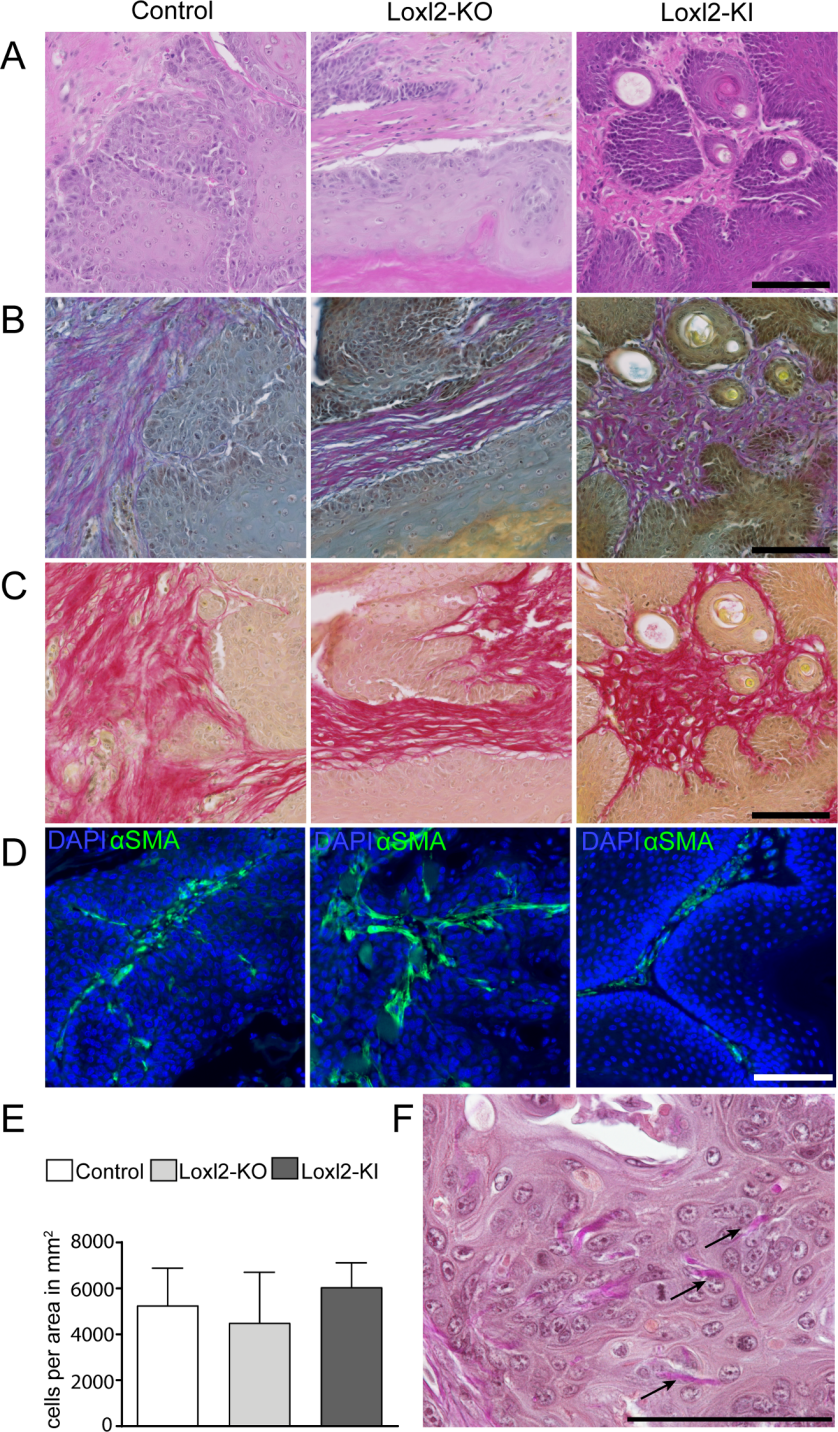
Loxl2 overexpression or deletion does not influence the tumour stroma composition of DMBA/TPA-induced papilloma. A-C: Hematoxylin and eosin (H&E) staining (A), Herovici staining (B), Picrosirius red staining (C) of DMBA/TPA induced papillomas from Loxl2-KO, Loxl2-KI and control mice. D: Immunostaining for α-SMA (green) in papillomas from Loxl2-KO, Loxl2-KI and control mice. Nuclei are labelled with DAPI (blue). E: Quantification of tumour stroma cell density. The data shown are means ± SD; (n = 3 control, n = 2 Loxl2-KO, n = 2 Loxl2-KI). F: Transepidermal perforation in an SCC stained with Elastica van Gieson Staining. Representative image for all genotypes is shown. Scale bars: 100 μm.

It has been shown previously that *Loxl2* overexpression increases and *Loxl2* loss-of-function decreases skin tumour burden and malignant progression by affecting epidermal differentiation [12]. Based on these observations, we speculated that *Loxl2* might have an influence on the dermal tumour microenvironment. For this purpose, we analysed the stroma of DMBA/TPA-induced papillomas (n = 14 control, n = 2 Loxl2-KO, n = 10 Loxl2-KI) and squamous cell carcinomas (SCC) (n = 4 control, n = 2 Loxl2-KO, n = 1 Loxl2-KI) by H&E, Herovici and Picrosirius Red staining (Fig 6A–6C). The grade of differentiation of the tumours was comparable, regardless of genotype. There was no obvious difference in labelling for α-smooth muscle actin (α-SMA), a reported marker of cancer associated fibroblasts, or cell density in the stroma of papillomas from mice of different genotypes (Fig 6D and 6E).

While the stromal labelling differed to some extent between tumours and in different regions of the same tumour, we saw no consistent effects of *Loxl2* expression. In SCCs, we found prominent transepidermal elimination (perforation) of collagen fibres to a comparable extent in all genotypes (Fig 6F). Transepidermal elimination of collagen fibres was not observed in papillomas. Accordingly, we found no evidence that *Loxl2* expression affected the stroma of DMBA/TPA-induced tumours.

## Discussion

ECM assembly during dermal maturation involves crosslinking between collagen fibres to stabilise collagen microfibril structure [29]. How different Lox family members are involved in this process and influence skin dermal structure and mechanical properties is still poorly explored. Deregulated expression of LOXL2 has been implicated in the development of fibrosis and cancer in a variety of tissues and organs [3,14,30,31]. In human lung and liver fibrosis, LOXL2 is overexpressed and treatments with an inhibitory monoclonal antibody against LOXL2 were reported to be beneficial [32]. However, further studies in a large cohort of idiopathic lung fibrosis did not show a clinical benefit [33]. In light of divergent evidence on the therapeutic potential of targeting stromal LOXL2, it is important to elucidate the effect of LOXL2 modulation on tissue development and homeostasis.

In this study, we have examined the effect of *Loxl2* gain- or loss-of-function in the dermis. Surprisingly, while aberrant *Loxl2* expression severely affects the heart and testis [12], neither *Loxl2* loss- nor gain-of function influenced dermal development and cell density. In addition, there was no effect on the distribution of fibroblast subpopulations, fibroblast proliferation or apoptosis. There was no significant up- or down-regulation of other Lox family members, suggesting that no significant compensatory mechanisms were required for normal dermal maturation and homeostasis on mRNA level. In Loxl2-KI transgenics we could observe a slight increase in collagen remodelling activity with age. *Loxl2* expression did, however, influence dermis stiffness in adult mice.

The dermal changes induced by TPA occurred independently of genotype. This shows that LOXL2 is dispensable for TPA-induced epidermal hyperproliferation and skin inflammation. Thus, while in lung and liver LOXL2 has been identified as central regulator of tissue stiffness [34], this would appear not to be the case in skin. This would explain why LOXL2 inhibition as a treatment for fibrosis is not reported to affect the skin.

There was no effect of *Loxl2* expression on the stroma of DMBA/TPA-induced papillomas and SCCs. We speculate that the increased skin tumour burden in *Loxl2* gain-of-function mice is induced by LOXL2’s intracellular epidermal functions, such as inhibition of terminal differentiation, promoting epithelial-mesenchymal transition or altering epithelial cell polarity (14). Similarly, it has been shown recently that in breast cancer the pro-metastatic function of LOXL2 is independent of its role for ECM remodelling, since neither *Loxl2* ablation nor overexpression affected ECM stiffness or organisation in the metastatic and primary tumour sites [35]. In contrast, increased LOX secretion in the same tumour type drives matrix stiffening through collagen crosslinking, hereby enabling tumour cell dissemination and metastasis [36]. These observations underline the complex and paradoxical role of Lox family members in various types of cancer [31,37,38].

It has been shown recently that Lox family members are differentially expressed during wound healing in murine myofibroblasts. While *Loxl2* and *Loxl3* are significantly up regulated, expression of *Loxl4* is decreased, suggesting that different crosslinking enzymes are needed to stabilise the granulation tissue in a wound [39]. However, wound healing is not affected in the different *Loxl2* mice genotypes (F Salvador, A Martin & A Cano, unpublished observation), suggesting that LOXL2 is dispensable.

In summary, we have shown that *Loxl2* expression is not essential for dermal development, homeostasis or tumour stroma formation. Our study emphasises the need to further dissect the functions of LOX family members in order to develop successful LOX targeted treatment strategies for fibrosis and cancer [31].

## Supporting information

S1 Fig Expression of Lox family members in the dermis. A: RNA-sequencing results plotted as FPKM as published in the Hair-GEL library for Lox family members at P5 [40]. Epi = Epidermis, ORS = Outer Root Sheath, Mx = Matrix, MC = Melanocyte, DF = Dermal Fibroblast, DP = Total Dermal Papilla cells. The data shown are means ± SD. B,C: Genotyping of the Loxl2-KO (detected allele L2^-^) (B) and Loxl2-KI (detected allele R26^L2^) (C) mice and the corresponding controls (detected allele L2^lox^ and R26^+^). N = 2 biological replicates are shown.
(TIF)

S2 Fig Dermal proliferation and apoptosis are unaffected by Loxl2 deletion or overexpression. A: Cleaved caspase 3 (cCasp3) staining (red); B: Ki67 staining (red). Immunostaining for Itga6 (green) labels the basement membrane and nuclei are labelled with DAPI (blue). Scale bar: 100 μm. C: Quantification of percentage cCasp3^+^ (left panel) and Ki67^+^ (right panel) cells in the dermis. Single data points for each mouse are shown and plotted with the mean; n = 2 for P2 Loxl2-KI, n = 3 for all others.
(TIF)

S3 Fig Expression of fibroblast subpopulation markers with age. A,B: Mean immunofluorescence intensity quantification of SCA1 (A) and CD26 (B) in the upper and lower dermis (n = 2 for P2 Loxl2-KI, n = 3 for all others). Data are shown as means ±SD.
(TIF)

S4 Fig Collagen fibres in the extracellular matrix of Loxl2-KO and Loxl2-KI mice dermis are not changed upon deletion or overexpression of Loxl2. A,B: Picrosirius red staining of Loxl2-KO, Loxl2-KI and control skin samples at P2, P21 and P120 visualised in polarised light (A) and shown in binary images (B). C: Collagen fibre structure in the dermis was analysed by B-CHP-staining (green); sections were counterstained with DAPI (blue). Scale bars: 100 μm.
(PDF)

S1 Table List of human tissue samples. (DOCX)

S2 Table qPCR primers and probes. (DOCX)

S3 Table Genotyping primer for Loxl2-KO and Loxl2-KI mice. (DOCX)

S4 Table Primary antibody list. (DOCX)
