# Supplementary figures and images for: Loxl2 is dispensable for dermal development, homeostasis and tumour stroma formation
Source: PLoS One. 2018 Jun 28;13(6):e0199679. doi: 10.1371/journal.pone.0199679 (PMC6023175; doi:10.1371/journal.pone.0199679)

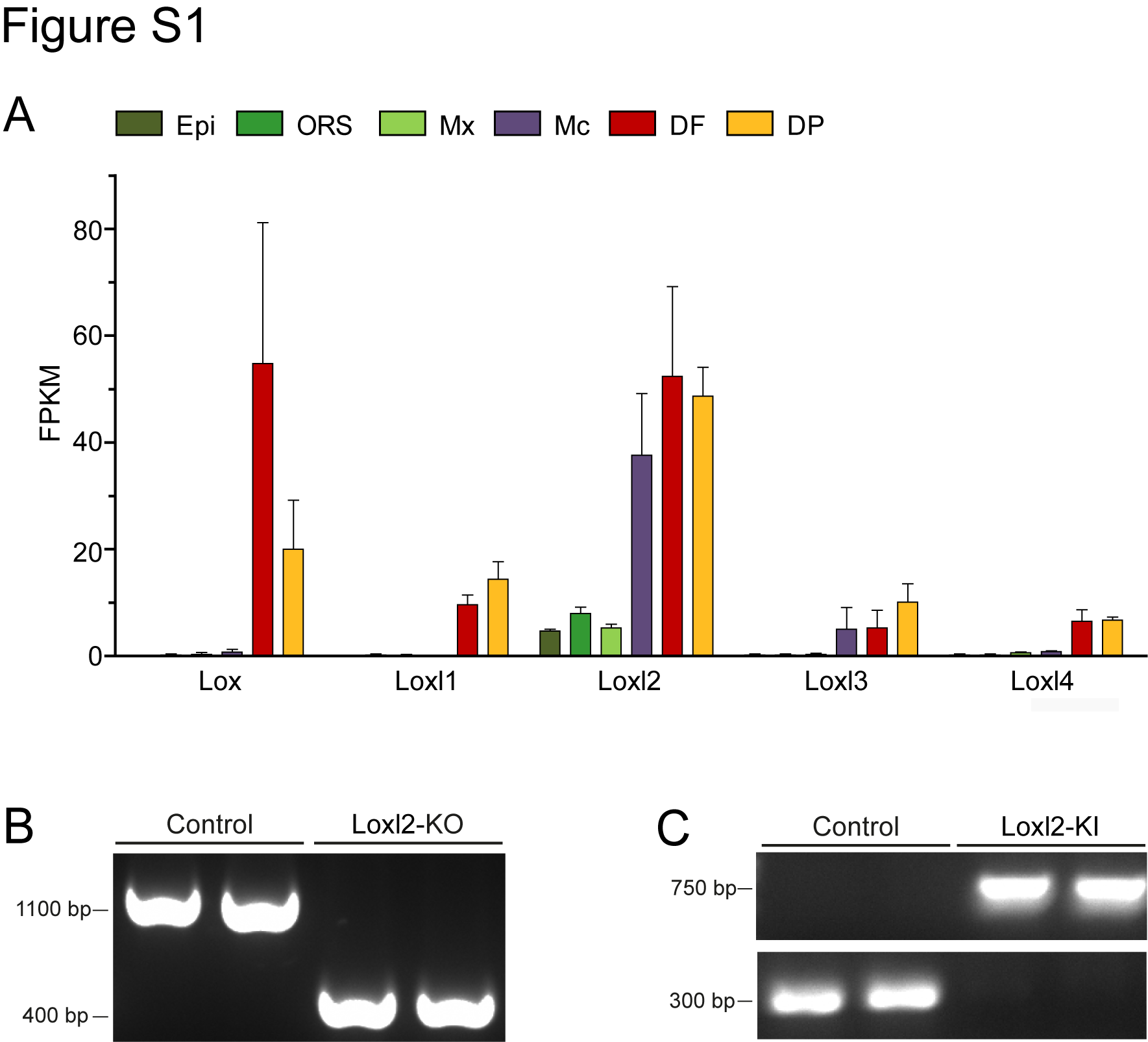

Supplement: S1 Fig — A: RNA-sequencing results plotted as FPKM as published in the Hair-GEL library for Lox family members at P5 [40]. Epi = Epidermis, ORS = Outer Root Sheath, Mx = Matrix, MC = Melanocyte, DF = Dermal Fibroblast, DP = Total Dermal Papilla cells. The data shown are means ± SD. B,C: Genotyping of the Loxl2-KO (detected allele L2-) (B) and Loxl2-KI (detected allele R26L2) (C) mice and the corresponding controls (detected allele L2lox and R26+). N = 2 biological replicates are shown. (TIF) [file pone.0199679.s001.tif]

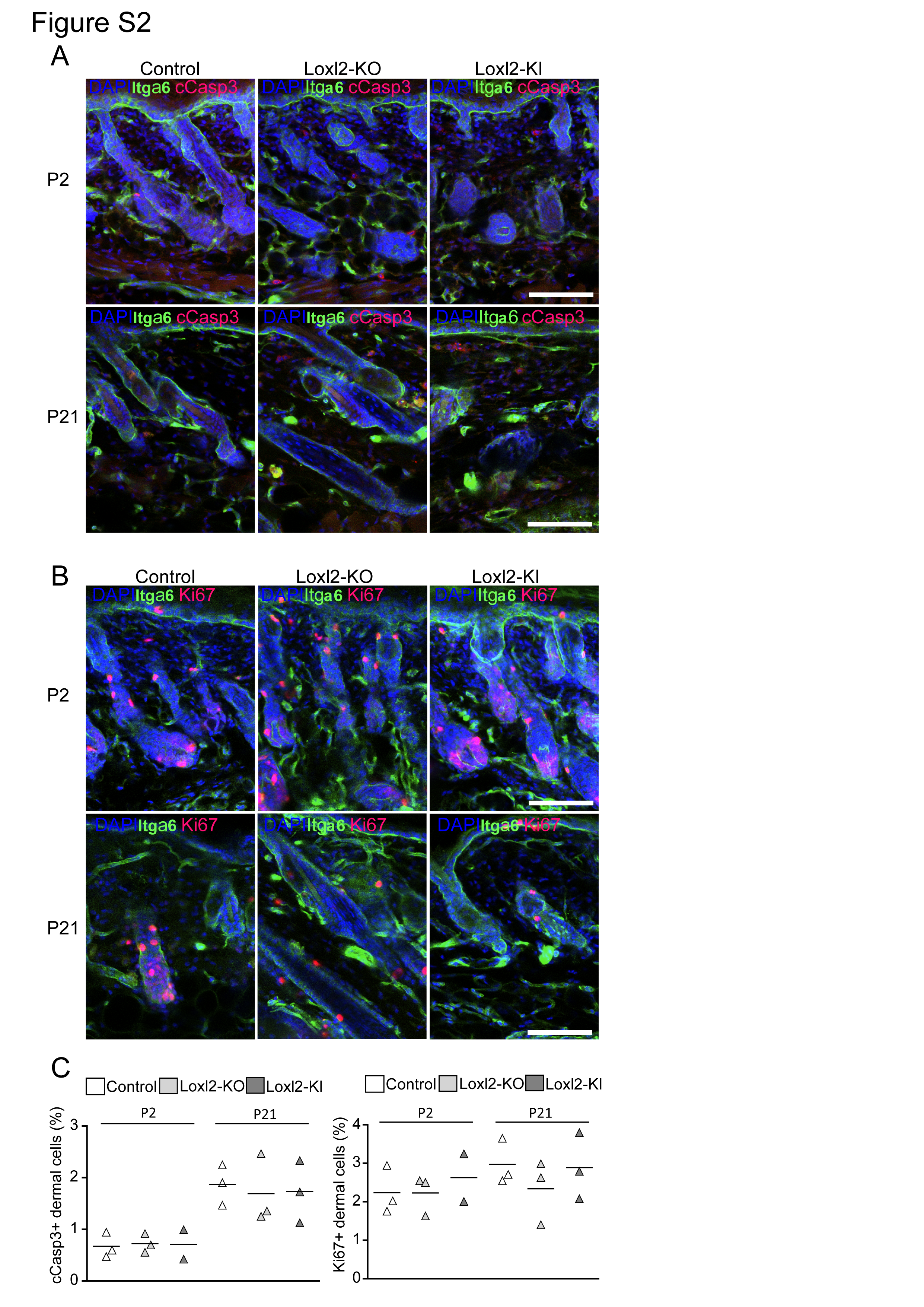

Supplement: S2 Fig — A: Cleaved caspase 3 (cCasp3) staining (red); B: Ki67 staining (red). Immunostaining for Itga6 (green) labels the basement membrane and nuclei are labelled with DAPI (blue). Scale bar: 100 μm. C: Quantification of percentage cCasp3+ (left panel) and Ki67+ (right panel) cells in the dermis. Single data points for each mouse are shown and plotted with the mean; n = 2 for P2 Loxl2-KI, n = 3 for all others. (TIF) [file pone.0199679.s002.tif]

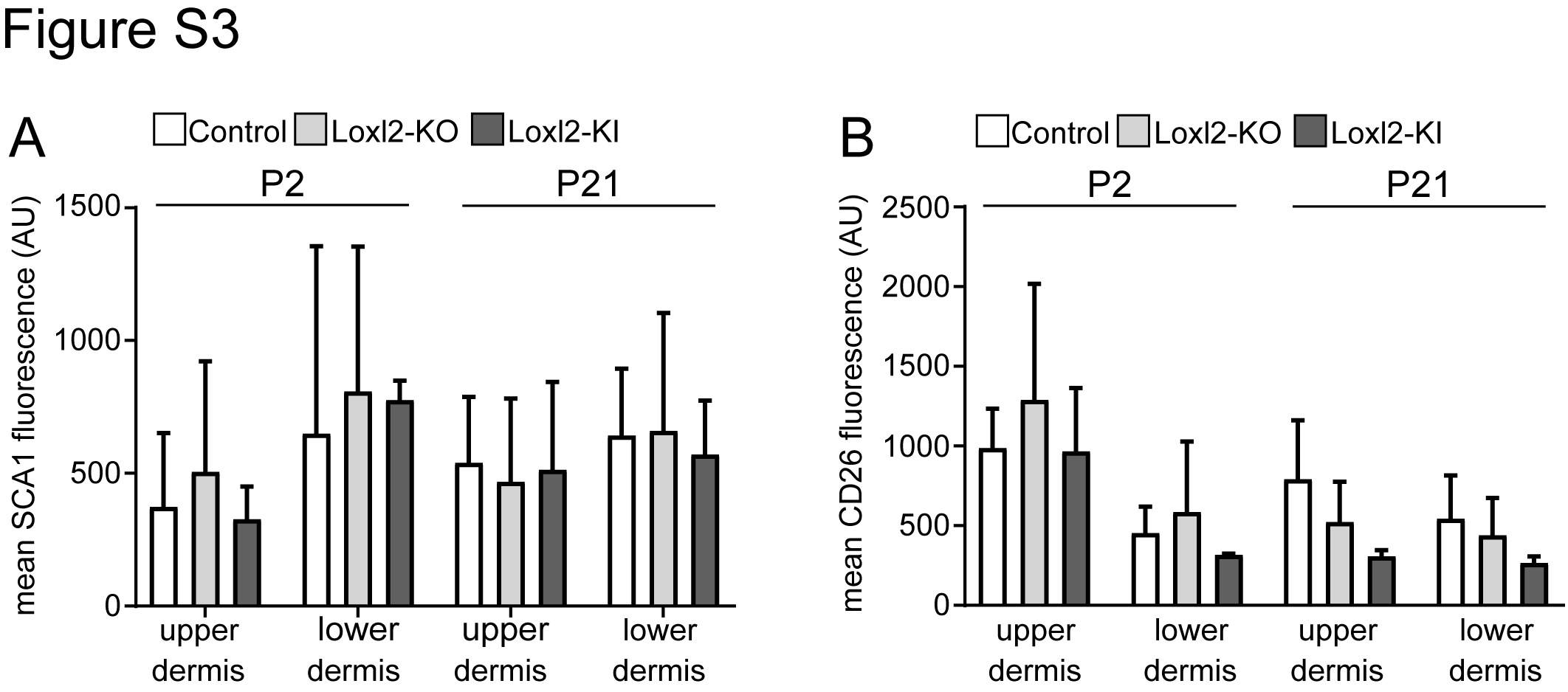

Supplement: S3 Fig — A,B: Mean immunofluorescence intensity quantification of SCA1 (A) and CD26 (B) in the upper and lower dermis (n = 2 for P2 Loxl2-KI, n = 3 for all others). Data are shown as means ±SD. (TIF) [file pone.0199679.s003.tif]

Figure S4

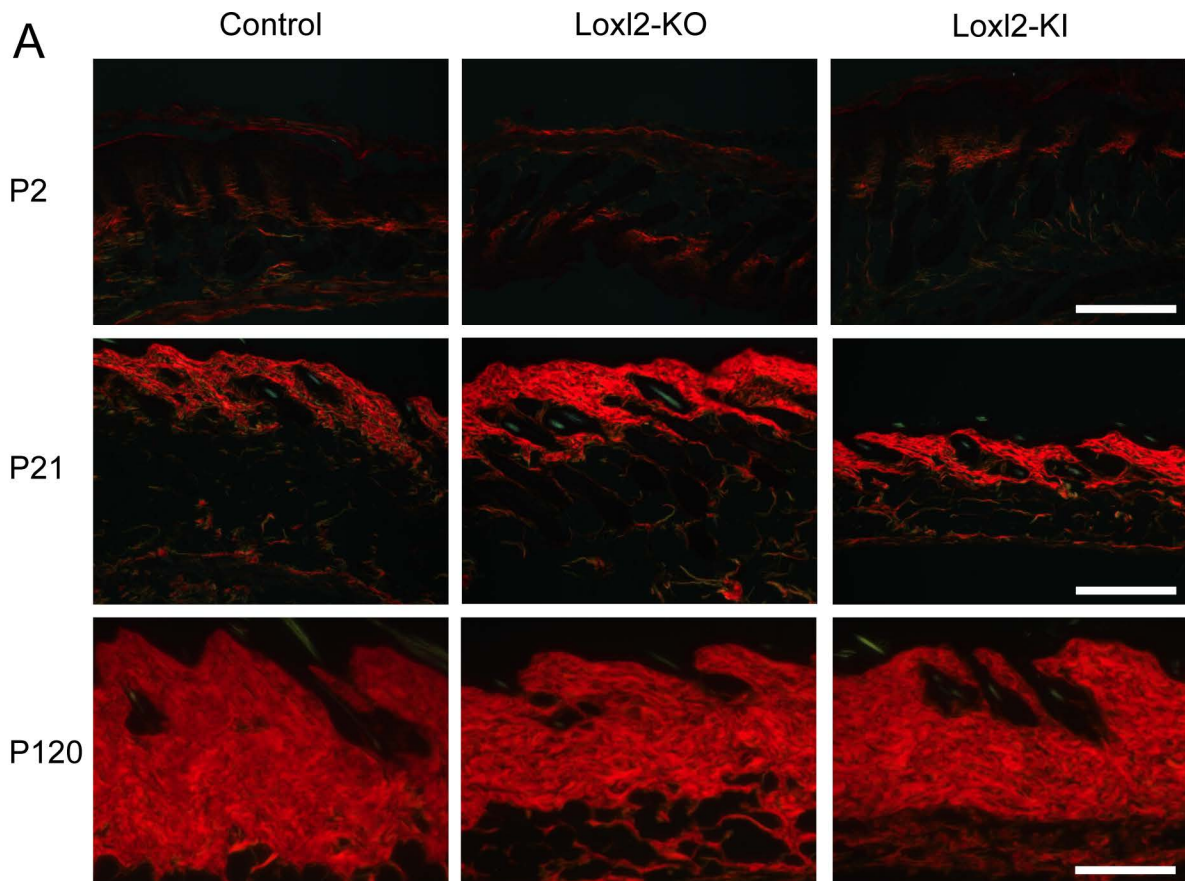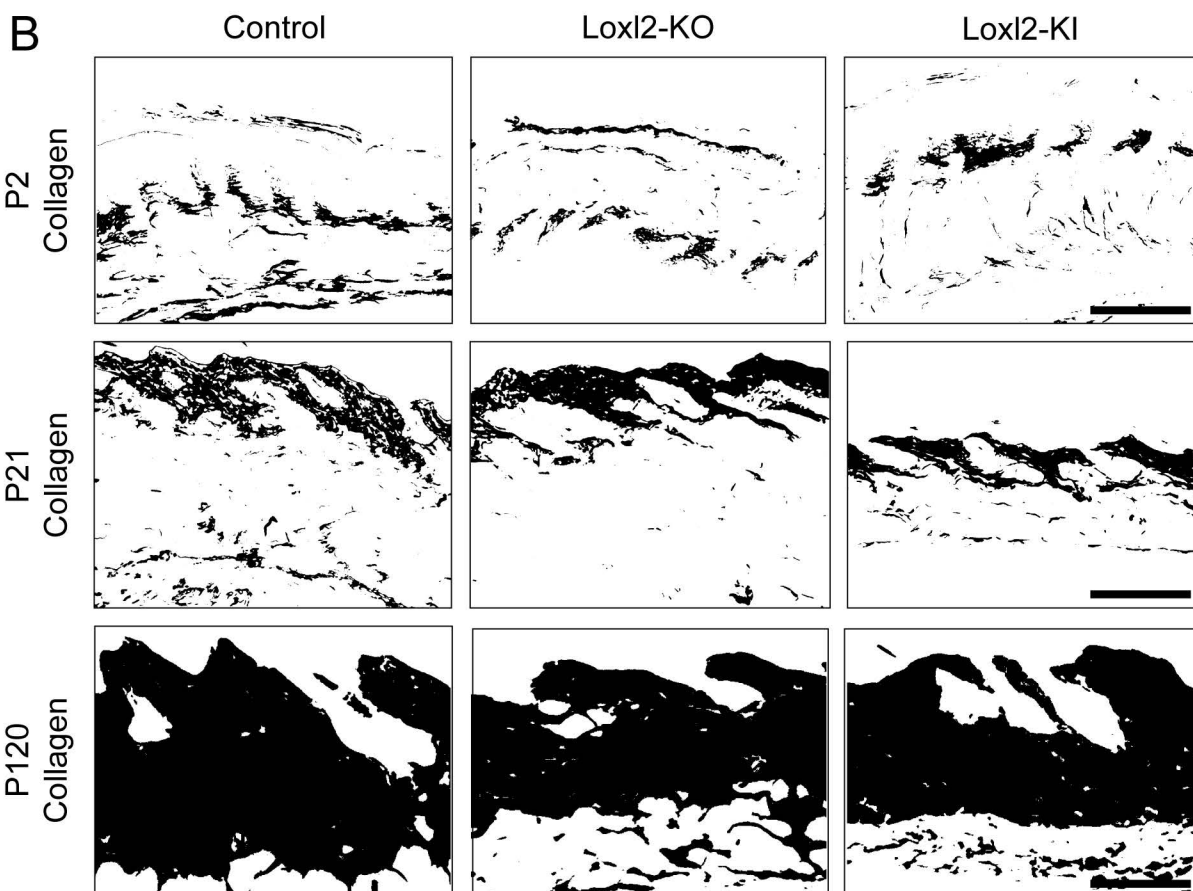

Figure S4

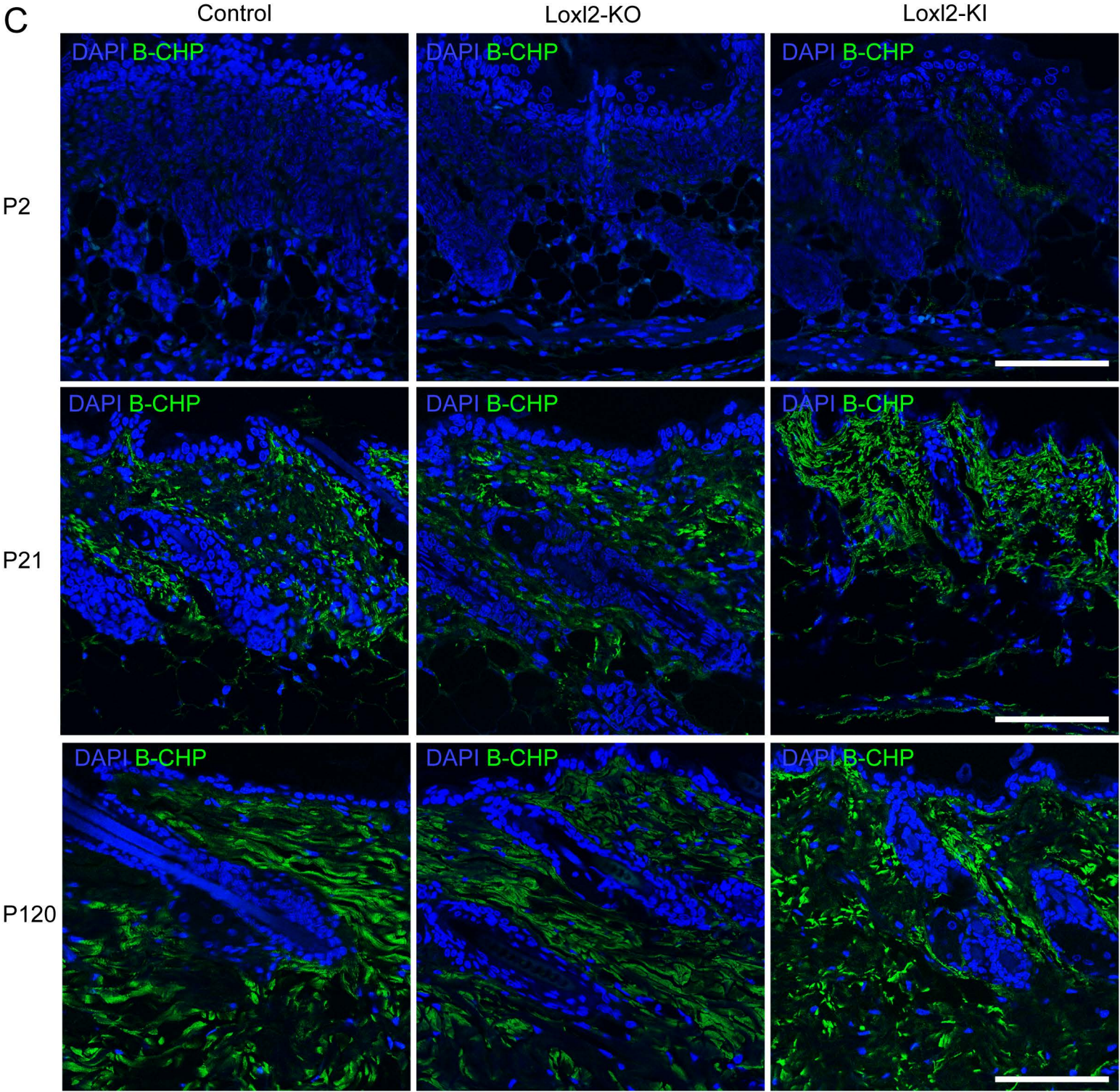

Supplement: S4 Fig — A,B: Picrosirius red staining of Loxl2-KO, Loxl2-KI and control skin samples at P2, P21 and P120 visualised in polarised light (A) and shown in binary images (B). C: Collagen fibre structure in the dermis was analysed by B-CHP-staining (green); sections were counterstained with DAPI (blue). Scale bars: 100 μm. (PDF) [file pone.0199679.s004.pdf]
