# Supplementary material for: Loxl2 is dispensable for dermal development, homeostasis and tumour stroma formation
Source: PLoS One. 2018 Jun 28;13(6):e0199679. doi: 10.1371/journal.pone.0199679 (PMC6023175; doi:10.1371/journal.pone.0199679)
Supplement: S1 Table — (DOCX) [file pone.0199679.s005.docx]

**S1 Table. List of human tissue samples.**

| **Sample** | **Age** | **Gender** | **Tissue location** |
| --- | --- | --- | --- |
| **Foetal** | **13 weeks** | **unknown** | **Back skin** |
| **Adult1** | **53 years** | **male** | **Abdomen** |
| **Adult2** | **45 years** | **female** | **Breast** |
| **Adult3** | **52 years** | **female** | **Breast** |
| **Adult4** | **62 years** | **female** | **Breast** |
