# Supplementary material for: Loxl2 is dispensable for dermal development, homeostasis and tumour stroma formation
Source: PLoS One. 2018 Jun 28;13(6):e0199679. doi: 10.1371/journal.pone.0199679 (PMC6023175; doi:10.1371/journal.pone.0199679)
Supplement: S2 Table — (DOCX) [file pone.0199679.s006.docx]

**S2 Table. qPCR primers and probes.**

| **Mouse primer and probes from Universal Probe Library (UPL, Roche)** | | | | |
| --- | --- | --- | --- | --- |
| **Gene** | **Primer sequence forward (5’-3’)** | **Primer sequence reverse (5’-3’)** | **UPL #** | **Efficiency** |
| Lox | caggctgcacaatttcacc | caaacaccaggtacggcttt | 48 | 2,01 |
| Loxl1 | tatgcctgcacctctcacac | tgtccgcattgtatgtgtcat | 64 | 1,98 |
| Loxl2 | cctacaaccccaaagcctataa | cgtgcagttcatagaaaacttcc | 69 | 2,03 |
| Loxl3 | ctgcaagaagacgggaacc | ttcctgtaccagtgccgagt | 84 | 1,92 |
| Loxl4 | gagtctggttgcacaactgc | ttgttcctgagtcgctgttc | 1 | 1,96 |
| Rn18S | gagaaacggctaccacatcc | gggtcgggagtgggtaat | 40 | 2,01 |
| Actb | tcctccctggagaagagcta | atgccacaggattccatacc | 27 | 1,98 |
|  |  |  |  |  |
| **Human primer (Sigma)** | |  |  |  |
| Lox | gtgctgctcagatttcccca | cctctgggtgttggcatcaa |  |  |
| Loxl1 | gcatgcacctctcataccca | gatgtagttcccaggctgca |  |  |
| Loxl2 | tgacgacttctccatccacg | gtgtgcttgcagtcagtgac |  |  |
| Loxl3 | tgatgacttcacgctgcagg | gggaggcacattcagtcaca |  |  |
| Loxl4 | atgaacaacagcagggtggt | tcctgtagtagtggctgtcga |  |  |
| RPL13A | aacagctcatgaggctacgg | aacaatggaggaagggcagg |  |  |
| TBP | gtgacccagcatcactgtttc | gagcatctccagcacactct |  |  |
