# Supplementary material for: Loxl2 is dispensable for dermal development, homeostasis and tumour stroma formation
Source: PLoS One. 2018 Jun 28;13(6):e0199679. doi: 10.1371/journal.pone.0199679 (PMC6023175; doi:10.1371/journal.pone.0199679)
Supplement: S3 Table — (DOCX) [file pone.0199679.s007.docx]

**S3 Table. Genotyping primer for Loxl2-KO and Loxl2-KI mice.**

| **Allele** | **Primer sequence forward (5’-3’)** | **Primer sequence reverse (5’-3’)** | **PCR product size (in bp)** |
| --- | --- | --- | --- |
| L2^+^ wild type | atcgggaattcagactgctg | gaggtgggcacagactaatc | 875 |
| L2^lox^ conditional |  |  | 1100 |
| L2^-^ knockout |  |  | 410 |
| R26^+^ wild type | tatcagtaagggagctgcagtg | accccagatgactacctatcctc | 300 |
| R26^L2^ overexpression | tatcagtaagggagctgcagtg | cccactggccttcgtagtagac | 750 |
