# Supplementary material for: Loxl2 is dispensable for dermal development, homeostasis and tumour stroma formation
Source: PLoS One. 2018 Jun 28;13(6):e0199679. doi: 10.1371/journal.pone.0199679 (PMC6023175; doi:10.1371/journal.pone.0199679)
Supplement: S4 Table — (DOCX) [file pone.0199679.s008.docx]

**S4 Table. Primary antibody list.**

| **Target** | **Species** | **Dilution** | **Company** | **Type** | **Application** | **Cat. -No.** |
| --- | --- | --- | --- | --- | --- | --- |
| CD45 | **Rat** | **1:500** | eBioscience | Clone 30-F11 | FACS | 17-0451-82 |
| CD31 | **Rat** | **1:500** | Biolegend | Clone 390 | FACS | 102410 |
| CD324 | **Rat** | **1:500** | eBioscience | Clone DECMA-1 | FACS | 50324982 |
| **CD26** | **rat** | **1:200** | eBioscience | **Clone H194-112** | **FACS** | 45-0261 |
| **Sca1** | **rat** | **1:200** | eBioscience | **Clone D7** | **FACS** | 12-5981-82 |
| **CD49f/ ITGA6** | **Rat** | **1:500** | **Biolegend** | **Clone GoH3** | **IF** | 31360 |
| **cCASP3** | **Rabbit** | **1:500** | R&D Systems | **polyclonal** | **IF** | AF835 |
| **KI-67** | **Rabbit** | **1:500** | **abcam** | **Polyclonal** | **IF** | 137802 |
| **ASMA** | **Rabbit** | **1:500** | **abcam** | **Polyclonal** | **IF** | **ab5694** |
| **DLK1** | **Goat** | **1:500** | R&D Systems | **polyclonal** | **IF** | AF1144 |
| **CD26** | **Rat** | **1:500** | **Biolegend** | **H194-112** | **IF** | **137802** |
| **LRIG1** | **Goat** | **1:500** | R&D Systems | **polyclonal** | **IF** | AF3688 |
| **SCA-1** | **Rat** | **1:500** | **BD Pharmingen** | **E13-161.7** | **IF** | **553333** |
